# Editorial: WW Domain Proteins in Signaling, Cancer Growth, Neural Diseases, and Metabolic Disorders

**DOI:** 10.3389/fonc.2019.00719

**Published:** 2019-08-02

**Authors:** Nan-Shan Chang, Rongtuan Lin, Chun-I Sze, Rami I. Aqeilan

**Affiliations:** ^1^Institute of Molecular Medicine, National Cheng Kung University College of Medicine, Tainan, Taiwan; ^2^Department of Medicine, Lady Davis Institute-Jewish General Hospital, McGill University, Montreal, QC, Canada; ^3^Department of Cell Biology and Anatomy, National Cheng Kung University College of Medicine, Tainan, Taiwan; ^4^Faculty of Medicine, The Lautenberg Center for Immunology and Cancer Research, Institute for Medical Research, Israel-Canada (IMRIC), Hebrew University of Jerusalem, Jerusalem, Israel

**Keywords:** WW domain, WWOX, signaling, Hippo, Smurf, neurodegeneration, cancer

First of all, the editorial team welcomes you to the specific Research Topic on “WW Domain Proteins in Signaling, Cancer Growth, Neural Diseases, and Metabolic Disorders.” We appreciate the hard work and outstanding contributions from all authors. WW domain is well-known for its participation in mediating protein-protein interactions, especially its role in relaying many signaling cascades. WW domains mediate these interactions through recognition of proline-rich peptide motifs and phosphorylated serine/threonine-proline sites. They are found in many different structural and signaling proteins that are needed in a variety of cellular processes. In our recent analysis of the human proteome, there are at least 52 WW domain-containing proteins and more than 10,000 among all species that play various roles in vital cellular processes (1). Dysregulation of WW domain-mediated signaling cascades disrupts the normal physiology and results in disease states. Indeed, WW domain proteins and their binding-partner complexes have been implicated in major human diseases including cancer, neural diseases and metabolic disorders. For instance, WW-domain proteins YAP and TAZ of the Hippo pathway participate in the regulation of cell stemness maintenance, tissue homeostasis, and tumorigenesis, thus making them targets for new diagnostics and therapeutics (Chen et al.). Importantly, tumor suppressor *WWOX* gene has recently been recognized as one of the five new risk factors in Alzheimer's disease (2).

Yet, our understanding and the fundamental knowledge of the entire WW domain family proteins are very limited. This has prompted us to propose to the *Frontiers* journals a specific thematic issue discussing recent knowledge and advancement on WW domain proteins in physiology and diseases. Prior to this, we have launched a thematic issue on tumor suppressor WWOX (WW domain-containing oxidoreductase) that was published in *Experimental Biology and Medicine* in 2015. Over the recent few years, wakeup calls from parents of newborn patients with WWOX deficiency have pushed us to propose this specific issue. These ill-fated little angels suffer from severe neural diseases which unfortunately still have no cure. Our efforts, which we dedicate to WWOX patients and their parents, aim to enrich our discussion about this important topic and brainstorm new venues to help fight related diseases.

What's new? We will feature articles in the WW domain-regulated signal pathways, and then present articles dealing with WWOX in physiology and diseases. First of all, Koganti et al. reviewed the inhibitory Smurf family proteins for the bone morphogenetic protein (BMP) and the transforming growth factor beta (TGF-β) signaling pathways and addressed their crucial roles in cancer progression. As a C2-WW-HECT E3 ligase, Smurf1 is an oncogenic protein, whereas Smurf2 acts as a tumor suppressor and oncogenic protein. The oncogenic function of Smurf2 is due to its stabilization of KRAS, EGFP and upregulation of Wnt/β-catenin pathway. Smurf proteins in cancer cell migration, metastasis and autophagy are also described. Next, in a related pathway, Chen et al. reviewed the ubiquitous feature of the Hippo signal pathway for organ development, with special focus on the WW domain proteins YAP and TAD. Dysregulation of the Hippo signal pathway leads to organ outgrowth and cancer progress (Chen et al.). In physiological settings, YAP and TAZ orchestrate the embryonic development, organ growth, tissue regeneration, stem cell pluripotency, and tumorigenesis. Chen et al. addressed the crucial role of YAP/TAZ in balancing the stem cell niches, which is important for normal development, as well as cancer progression. Supporting research from WWOX also shows this protein may oversee the Hippo signaling pathway from the upstream via interacting with proteins in the TGF-β, hyaluronidase Hyal-2, and Wnt/β-catenin pathways (Chen et al.). In supporting this notion, a recent study reported that downregulation of WWOX results in tamoxifen resistance in breast cancer due to inactivation of Hippo signaling (3). Lee and Liou described the structure and the functional nature of Pin1. As a family of the peptidyl-prolyl *cis*-*trans* isomerase (PPIase), Pin1 catalyzes the *cis*/*trans* isomerization of the proline residue in the phosphorylated Serine/Threonine-Proline (S/T-P) motifs of substrates. The WW domain of Pin1 preferentially binds numerous protein substrates possessing the *trans* configuration of the phosphorylated S/T-P motif, which are needed in cell events such as cell cycle, transcription, DNA damage, and apoptosis. The PPIase catalyzes the *cis* to *trans* isomerization, whereas this may hinder WW domain in binding substrates.

Regarding the WWOX area, Jamous and Salah reviewed the role of WWOX and other WW domain proteins in breast cancer tumorigenesis. Similarly, Pospiech et al. described the history of WWOX research and association with breast cancer progression. Tanna and Aqeilan discussed the use of animal models to assess *in vivo* WWOX functions. The review covers the rodent, fish, and fly models. Defects in growth retardation, metabolism, reproduction, neural system, and early death are discussed. Saigo et al. reviewed the inhibitory proteins for WWOX, specifically with TMEM207. The WW domain of WWOX binds the PPxY motif in TMEM207. TMEM207 contribution to the pathogenesis of cancer was discussed. Hussain et al. utilized experimental approaches and identified WWOX-binding proteins. WWOX interactors are associated with metabolic pathways for proteins, carbohydrates, and lipids breakdown. In supporting the role of WWOX in maintaining DNA stability, McBride et al. reported *Wwox* deletion in mouse B cells leads to the development of genomic instability, neoplastic transformation, and monoclonal gammopathies. While loss of WWOX in newborns leads to severe neural diseases and early death, Liu et al. reviewed the cascade of WWOX downregulation-induced protein aggregation that causes neurodegeneration. Additionally, switch of the phosphorylation of WWOX at Tyr33 for anticancer response to Ser14 for disease progression (e.g., cancer and AD) is discussed. Suppression of Ser14 phosphorylation by a zinc finger peptide Zfra blocks cancer growth and restores memory loss in mice (4, 5).

Finally, what's urgent for the field? It would be of great importance to have a cure for the newborn patients who suffer severe neural diseases due to WWOX deficiency, and provide a complete termination for the severe progression of neurodegeneration in AD patients. For example, an effective drug to lessen seizure in the newborn patients would greatly benefit them. Preliminary findings from clinical treatment shows that despite mutations, forced transcription of *WWOX* gene appears to be a feasible approach to lessen the symptoms of seizure in patients with neurodegeneration (personal communications with Dr. D. S. Lin at the Taipei Medical University). Furthermore, blocking the downregulation of WWOX in the middle aged individuals would likely to prevent the development of AD.

In the concluding remarks, it is achievable to design WWOX-targeted therapy. Surface-enhanced Raman scattering (SERS) amplified Raman spectroscopy signal can be used for detecting and imaging biological specimens *in vitro* or *in vivo*. A recent success has utilized EGFR antibody to design Raman tags to target amplified EGFR in glioblastoma cells (6). By the same token, Raman tags can be designed to identify WWOX expression and its phosphorylation in normal neurons and glioblastoma cells in the brain, thus facilitating imaging, diagnosis, and treatment. However, there are expected difficulties in the brain imaging for patients, which requires further technical innovations. Small molecules such as synthetic chemicals or peptides can directly support treating patients in clinics, once they are functionally validated and approved for clinical use. Zfra peptides can be used as therapeutic options and strategies to target cancer and neural diseases associated with WWOX deficiency (4, 5).

## Author Contributions

N-SC initiated writing the original manuscript, revised, proof read, discussed with co-authors, and finalized the manuscript. RL and C-IS read, revised and proofed the manuscript. RA contributed in part to writing, revised and proofed the manuscript.

### Conflict of Interest Statement

The authors declare that the research was conducted in the absence of any commercial or financial relationships that could be construed as a potential conflict of interest.
